# Natural Coronary Bypass: A Rare Case of Aortocoronary Fistula in a Patient with Three-Vessel Disease

**DOI:** 10.7759/cureus.7023

**Published:** 2020-02-17

**Authors:** Syed Arsalan Ahmed Naqvi, Syed Danish Zaidi, Owais Gul, Mudassir Iqbal Dar, Abdul Aziz

**Affiliations:** 1 Internal Medicine, Dow University of Health Sciences, Karachi, PAK; 2 Cardiac Surgery, Civil Hospital Karachi, Karachi, PAK; 3 Internal Medicine, Jinnah Medical and Dental College, Karachi, PAK

**Keywords:** coronary arterial fistula, aortocoronary fistula, coronary artery bypass grafting

## Abstract

Coronary arterial fistula (CAF) is an abnormal connection between one or both coronary arteries and a cardiac/extra-cardiac chamber or another vessel. Aortocoronary fistula is a rare type of CAF, which involves an anomalous connection between coronary arteries and any segment of aorta. The following case report describes the case of an adult male patient who presented with the complaint of typical chest pain. Coronary angiography was done and the diagnosis of severe three-vessel disease with an associated aortocoronary fistula was made. Coronary artery bypass grafting (CABG) was planned and performed, and the patient was discharged after a week postoperatively.

## Introduction

Coronary arterial fistula (CAF) is a rare anomaly that involves an abnormal connection between one or more coronary arteries and either a heart chamber (known as coronary-cameral fistula) or a systemic/pulmonary vessel (known as coronary-arteriovenous fistula) [1]. Most patients are asymptomatic while others present with typical chest pain and classical manifestations of angina pectoris or myocardial infarction secondary to thromboembolic events, coronary steal phenomenon or concomitant coronary artery stenosis [2]. We describe the case of a 55-year-old man who presented with typical chest pain due to underlying severe three-vessel coronary artery disease, with an associated aortocoronary fistula, which was diagnosed on performing coronary angiography.

## Case presentation

A 55-year-old married man presented to our outpatient clinic with the primary complaints of chest pain and exertional shortness of breath (SOB) for three months. He had been experiencing chest pain while performing routine activities. The pain was non-radiating, dull and was relieved by rest. SOB was equivalent of New York Heart Association (NYHA) class II grade. The patient did not report any pedal edema, orthopnea, paroxysmal nocturnal dyspnea, palpitation, nausea, vomiting, excessive sweating, fever, joint pain, or trauma of any sort. He denied any history of congenital heart disease, congestive cardiac failure, prior myocardial infarction, diabetes mellitus, and hypertension. His systemic review was unremarkable. Past medical and surgical history was not significant. There was no history of smoking, alcohol, or recreational drug use. However, his family history was significant for cardiovascular disease with both of his parents dying due to myocardial infarction.

**Figure 1:**
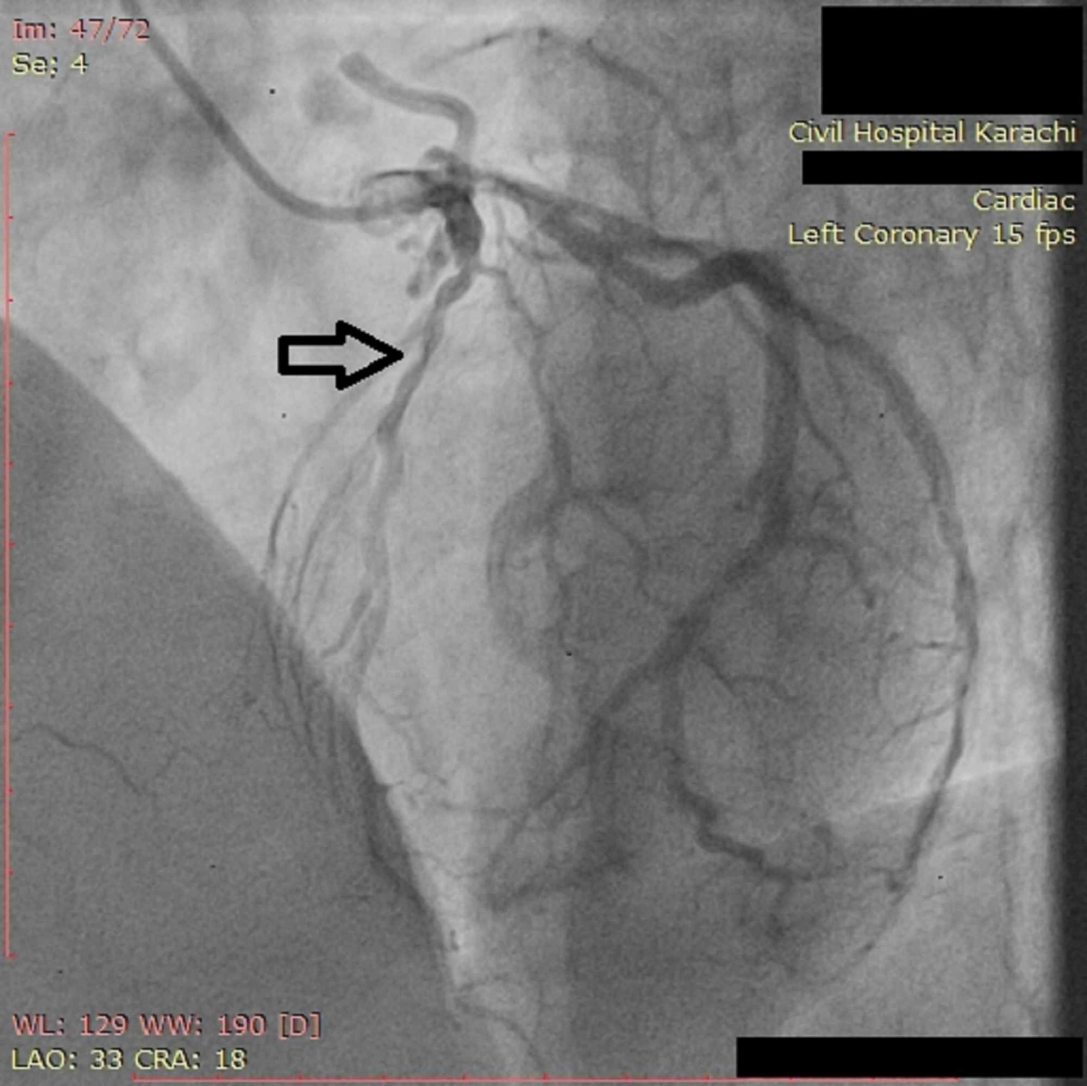
Angiogram of LAD coronary artery showing severe (70%) mid segmental stenosis LAD: Left anterior descending

**Figure 2:**
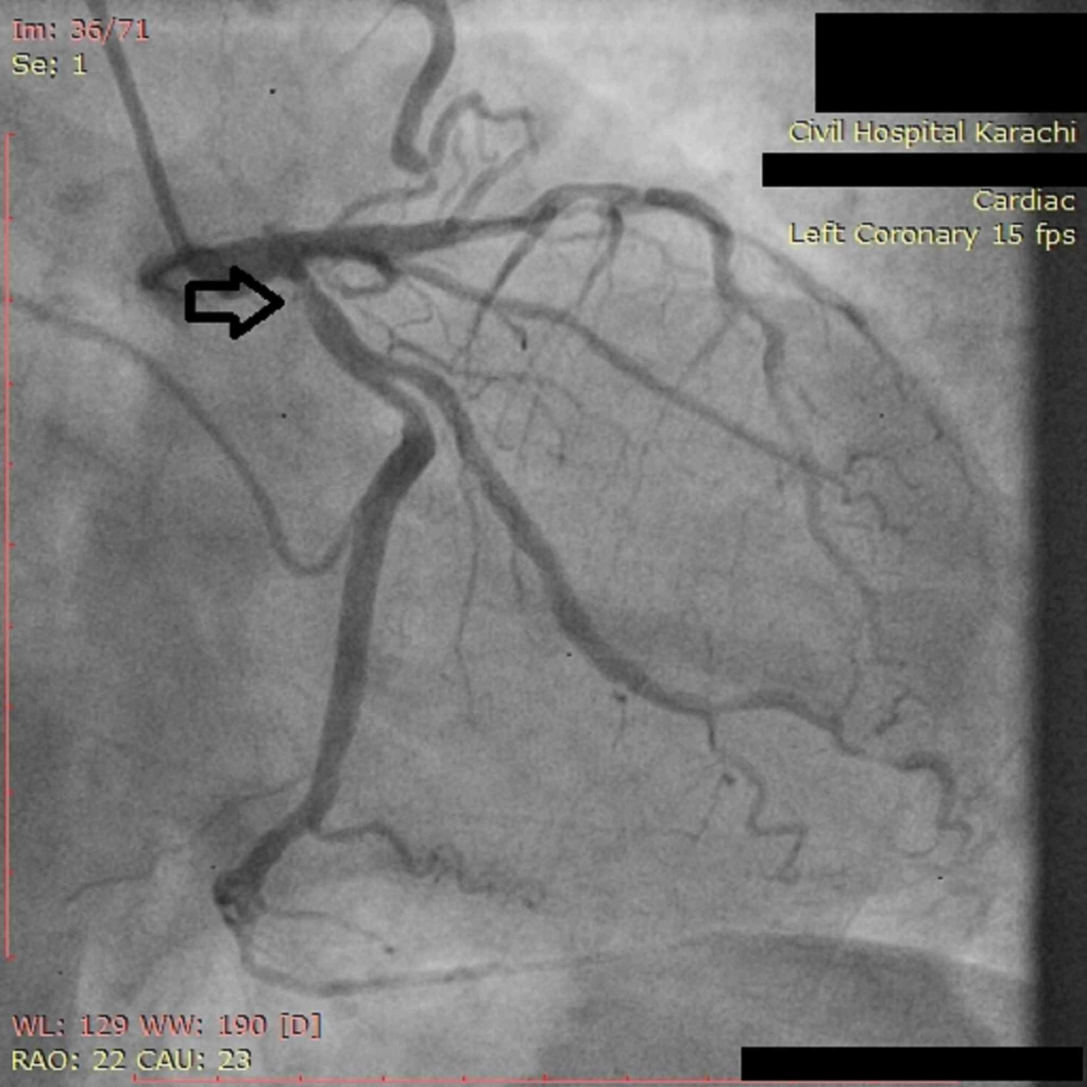
Angiogram of LCX coronary artery showing severe (70%) ostial stenosis LCX: Left circumflex artery

**Figure 3:**
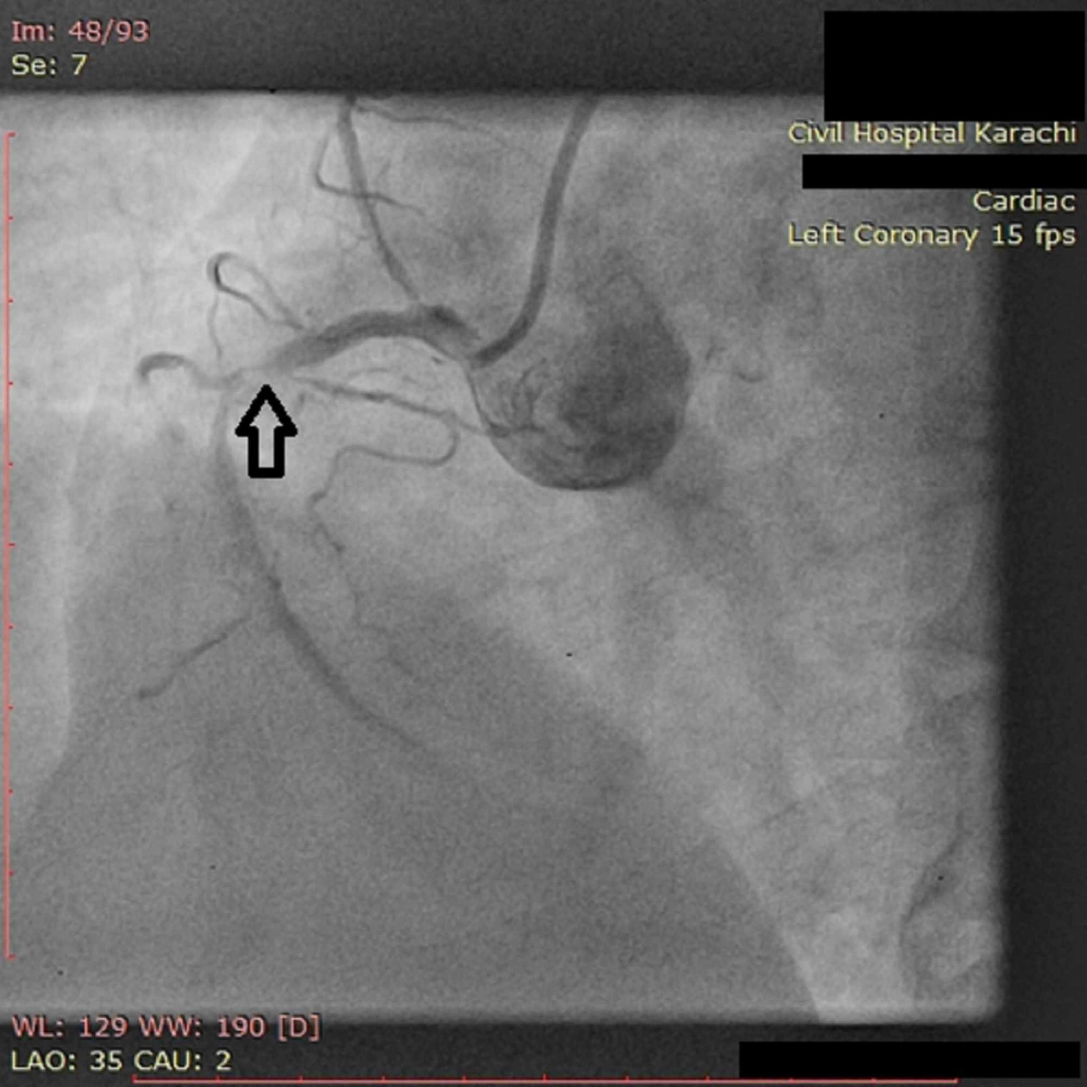
Angiogram of RCA showing severe (70%) distal segment stenosis RCA: Right coronary artery

**Figure 4:**
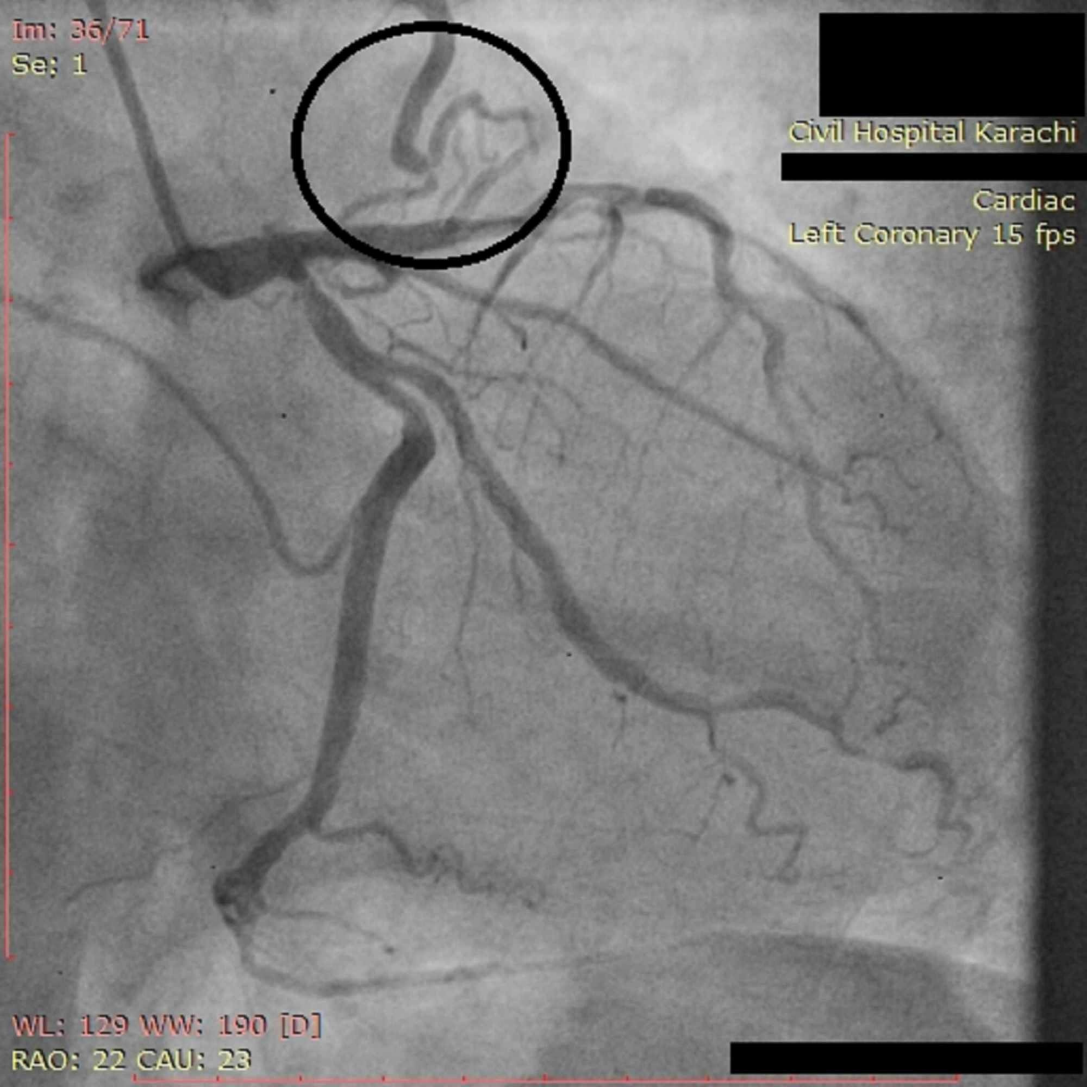
Angiogram showing small sized aortocoronary fistula

On physical examination, the patient was afebrile with a regular pulse rate of 66 beats per minute, a respiratory rate of 22 breaths per minute, and a blood pressure of 109/55 mmHg (millimeter of mercury). Cardiovascular examination revealed normal heart sounds without any murmurs. Lungs were clear on auscultation with equal air entry and no added sounds. The nervous and abdominal examinations were unremarkable. Electrocardiogram showed sinus rhythm. The patient was admitted for further investigations. Cardiac biomarkers including cardiac troponin-T (cTnT) and troponin-I (cTnI) were normal. Echocardiography revealed mild mitral regurgitation, mild left ventricular systolic and diastolic dysfunction, and segmental wall motion abnormality. Cardiac angiography was done via the right radial artery approach which revealed severe (70%) diffuse mid-segmental stenosis in the left anterior descending (LAD) artery, severe (70%) ostial stenosis of left circumflex (LCX) artery, and severe (70%) proximal and distal segment stenosis in the right coronary artery (RCA) (Figure 1-3). We were also able to identify a unilateral aortocoronary fistula that originated from the proximal LAD coronary artery and terminated into the proximal ascending aorta (Figure 4). The patient was admitted for coronary artery bypass (CABG) surgery. 

A conventional midline median sternotomy incision was used for on-pump CABG. Beating heart surgery equipment was also arranged and ready to be used in case of failed achievement of cardioplegia. Intermittent antegrade cold blood cardioplegia at a total dose of 350 milliliters was given for myocardial protection during aortic cross-clamping. The aorta was cross-clamped 7 cm from the aorto-ventricular junction. The left internal mammary artery (LIMA) and greater saphenous vein (GSV) were harvested for grafting. LIMA was grafted to the LAD artery, while GSV was grafted to the obtuse marginal and posterior descending artery. The aortic cross-clamp time (XCT) was 90 minutes and cardio-pulmonary bypass time was 112 minutes. Bilateral pleural drains were placed and a temporary pacing wire was inserted into the right ventricular free wall. The sternum was closed by steel wires wrapped around in an interrupted manner. The patient was extubated within 4 hours after surgery. Ionotropic support along with vasodilators was used to prevent low output. Cardiac rehabilitation was started. On the fourth postoperative day, the patient was shifted to the ward from the intensive care unit (ICU) and was discharged on the seventh postoperative day without any complications or active complaints.

## Discussion

CAF is a rare anomaly, which is mostly congenital in origin but can be acquired after trauma or invasive cardiac procedures such as valvular replacement, CABG surgery, percutaneous coronary intervention, and cardiac biopsies. In some cases, fistulas were found to be associated with Takayasu’s arteritis and other cardiac/systemic diseases. The exact incidence of CAF is unknown, but it has been reported to be less than 1% in several studies [3].

Likewise, aortocoronary fistula is an abnormal connection between one or both of the coronary arteries and any segment of aorta. A fistula between the aorta and the coronary artery is extremely rare and is most commonly seen after surgical interventions [4]. We here present a case of aortocoronary fistula in a patient with three-vessel disease. To the best of our knowledge, this is the first case reported from Pakistan.

Upon review of literature, we found a case reported by Sánchez-Soriano RM, et al., which showed an irregular aortocoronary fistula tract originating from right Valsalva sinus cranial to the ostium of RCA and terminating into the left main coronary artery with an associated ascending aortic aneurysm, and the absence of evident CAD in a 58-year-old male patient. The patient presented with typical chest pain for months, which usually occurred after emotional stress. Such patients, as opposed to our patient who had evident severe three-vessel disease, develop typical chest pain either due to coronary steal phenomenon or secondary endothelial dysfunction caused by high flow through the arteries. Coronary steal phenomenon is a mechanism by which a coronary artery fistula deprives the myocardium of its normal blood flow distal to the site of the anomalous connection, causing ischemia and subsequent chest pain [5].

Another case revealed an adult male patient who underwent CABG on a beating heart for severe three-vessel disease because cardioplegic arrest could not be achieved despite a total dosage of three liters. The failure to establish cardiac arrest was secondary to the presence of extra-cardiac collaterals and an aortocoronary fistula, which were causing severe backflow. This led to an increased post-operative dependence on extra-corporeal life support and the placement of an intra-aortic balloon pump (IABP) to recover from ventricular fibrillation and myocardial stunning [6]. Although the presence of an aortocoronary fistula can be protective in patients with CAD, it can complicate CABG to a significant extent, even in patients with a low-risk profile [7]. In contrast, the cardioplegic arrest in our patient occurred at a dose of 350 milliliters suggesting a small fistula with negligible backflow.

Coronary angiography and multi-detector computed tomography (MD-CT) scan, are used to visualize and diagnose CAF. MD-CT is a non-invasive modality, which provides a three-dimensional view of anomalous coronary arteries and their relationship with the surrounding structures, which aid in accurate diagnosis [3,8]. Furthermore, in cases of CAD proximal to the origin of CAF, the MD-CT scan is more reliable than coronary angiography to reveal the fistula tract [6]. However, in cases where the fistula tracts are not visible within the spatial resolution of the CT scan, coronary angiography is done as it is found to be more appropriate for such cases [5]. The surgical treatment of patients with CAF who are asymptomatic depends upon several factors such as age, anatomy, and risk of complications (thrombosis, aneurysm, rupture). Moreover, patients with symptoms can be treated with percutaneous transcatheter closure (TCC). Open heart surgery is opted only in patients with concurrent CAD or a large fistula with inconclusive anatomy [3].

## Conclusions

CAF is a rare anomaly, which can be congenital or acquired. Most patients are asymptomatic, but some patients may present with typical chest pain. Aortocoronary fistula can be protective in patients with concomitant CAD; however, it can complicate CABG depending upon its size and amount of backflow. MD-CT scan or coronary angiography are the diagnostic modalities used for accurate delineation of patient's coronary arterial anatomy in case of suspected CAF.
